# Toxicity of Per- and Polyfluoroalkyl Substances and Their Substitutes to Terrestrial and Aquatic Invertebrates—A Review

**DOI:** 10.3390/toxics13010047

**Published:** 2025-01-09

**Authors:** Jiaxin Zhang, Hassan Naveed, Keping Chen, Liang Chen

**Affiliations:** 1 School of Life Sciences, Jiangsu University, Zhenjiang 212013, China; jiaxinzhang2022@163.com (J.Z.); hassan.naveed88@outlook.com (H.N.); kpchen@ujs.edu.cn (K.C.); 2 School of Food and Biological Engineering, Jiangsu University, Zhenjiang 212013, China

**Keywords:** perfluoroalkyl substances, polyfluoroalkyl substances, invertebrates, toxicity, oxidative stress, neurobehavioral toxicity, developmental toxicity, reproductive toxicity

## Abstract

Per- and polyfluoroalkyl substances (PFASs) have been widely used in daily life but they cause certain impacts on the environment due to their unique carbon–fluorine chemical bonds that are difficult to degrade in the environment. Toxicological studies on PFASs and their alternatives have mainly focused on vertebrates, while terrestrial and aquatic invertebrates have been studied to a lesser extent. As invertebrates at the bottom of the food chain play a crucial role in the whole ecological chain, it is necessary to investigate the toxicity of PFASs to invertebrates. In this paper, the progress of toxicological studies on PFASs and their alternatives in terrestrial and aquatic invertebrates is reviewed, and the accumulation of PFASs, their toxicity in invertebrates, as well as the neurotoxicity and toxicity to reproduction and development are summarized. This provides a reference to in-depth studies on the comprehensive assessment of the toxicity of PFASs and their alternatives, promotes further research on PFASs in invertebrates, and provides valuable recommendations for the use and regulation of alternatives to PFASs.

## 1. Introduction

With the rapid development of modern industry, the number of pollutants is increasing, constantly threatening environmental safety, and PFASs are one of the new persistent organic pollutants. Per- and polyfluoroalkyl substances (PFASs) are organic compounds that contain at least one perfluorinated carbon atom. PFASs were born in the 1930s and include perfluorooctanoic acid (PFOA), perfluorooctane sulfonate (PFOS), and many other compounds. PFAS molecules have chains of carbon and fluorine atoms attached to the molecule, and because the carbon–fluorine bond is one of the strongest chemical bonds, they are chemically inert and resistant to high temperatures [1], which makes their products hydrophilic and lipophobic. They are widely used in consumer and industrial products that require grease or water repellency or surfactant action [2]. PFAS-related products include food packaging, non-stick cookware, cosmetics, water- and stain-resistant textiles and carpets, aqueous film-forming foams used to fight fires as part of the process, as well as corrosion inhibitors in aircraft hydraulic fluids, surfactants for electronic etching baths, photographic emulsifiers, waxes, paints, and adhesives [3,4,5].

**Figure 1:**
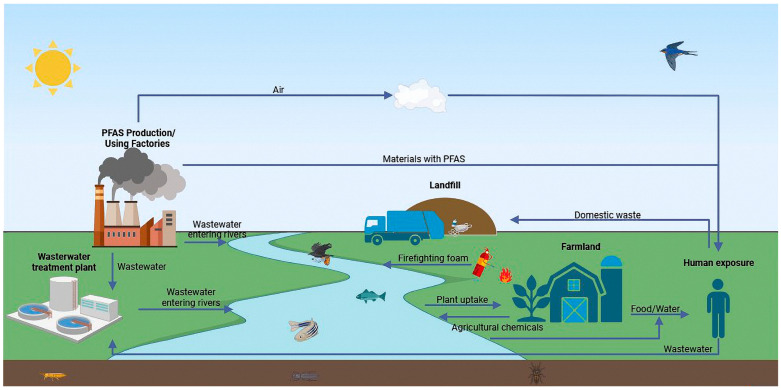
Map of the natural circulation of PFASs. PFASs have multiple dispersal pathways in the natural environment, such as industrial processes, wastewater treatment discharges, landfills, contaminated soils or water sources used in agriculture, and the redistribution of PFASs through volatilization, deposition, and leaching to air, soil, and water sources. These pathways can lead to long-term exposure of animals and humans to PFASs. In addition, PFASs accumulate through the food chain and are harmful to all organisms in the food chain.

The widespread use of PFASs has led to the ubiquitous presence of these chemicals in the environment, including rivers, soil, air [6], house dust, food, and drinking water from surface and groundwater sources [7]. PFASs are not readily degradable, and are often discharged directly into water bodies after use, further contaminating the soil. A variety of PFASs exist in the environment since PFASs are environmentally persistent, bioaccumulative, and biodegradation-resistant due to their strong carbon–fluorine bonds [8], and PFAS are able to disperse over long distances, even reaching the Antarctic and the Arctic [9,10,11,12]. They likewise bioaccumulate and amplify through the food chain [13,14,15,16,17], with significant implications for the safety of humans and wildlife. Various PFASs have been reported in human tissues and organs, blood [18], breast milk [19,20], etc., and they have been found to cause a variety of adverse effects on human health, which continue to increase (Figure 1).

Currently, PFASs are listed as hazardous under the Stockholm Convention on Persistent Organic Pollutants (POPs); therefore, short-chain PFASs such as perfluorobutanoic acid (PFBA), perfluorohexanoic acid (PFHxA), perfluorobutane sulfonic acid (PFBS) and perfluorohexanesulfonic acid (PFHxS) are no longer produced in many countries. PFHxS has emerged as a favorable alternative to PFOA and PFOS and has been widely used; however, the toxicological and epidemiological data are lacking, and there is evidence that new alternatives can lead to a variety of undesirable effects, such as lethality, growth inhibition, and reduced lifespan toxicity in invertebrates. Some substitutes are even more toxic than the original long-chain PFASs, and their mechanisms of action have not been clarified. Organisms are exposed to complex mixtures of PFASs in nature but most of the experimental results obtained so far have been obtained from exposure to a single contaminant, which may lead to some bias in assessing their overall risk. Intrinsic factors (gender, genetics, etc.) may contribute to differences in the toxicity of PFASs on organisms, and the interaction of PFASs with other factors in the environment, such as heavy metals, salinity, etc., may also modify adverse health outcomes. The safety of substitutes needs to be further investigated to obtain data for toxicity studies in different organisms.

Invertebrates are ubiquitous in natural ecosystems, where they are often at the bottom of the food chain and play a key role in material flow and energy cycling. Some invertebrates have become endangered due to the rise in human activities that have increased environmental pollution. Any decrease in invertebrate biomass will reduce the multifunctionality of ecosystems [21] and is crucial to studying the toxicity of PFASs in the environment.

To date, studies on PFASs have mainly focused on vertebrates but in recent years studies on invertebrates, especially terrestrial invertebrates, have been carried out. The aim of this paper is to summarize the toxicity of PFASs and their alternatives on invertebrates by means of a literature review, which will help to provide a more comprehensive understanding of PFASs in the natural world, and to more holistically assess the toxicity of PFASs and their alternatives.

## 2. Methods

A literature search was performed on PubMed and Google Scholar by the following keywords: perfluoroalkyl acids (PFAAs), perfluorinated compounds (PFC), Per- and polyfluoroalkyl substances (PFASs), perfluorooctanoic acid (PFOA), perfluorooctane sulfonate (PFOS), perfluorononanoic acid (PFNA), perfluorodecanoic acid (PFDA), perfluorododecanoic acid (PFDoA; PFDoDA), perfluorodecanesulfonic acid (PFDS), perfluorobutane sulfonic acid (PFBS), perfluoropentanoic acid (PFBA), perfluoroundecanoic acid (PFUnA; PFUnDA), perfluoro octanesulfonamide (PFOSA), perfluoroethylcyclohexane sulfonic acid (PFECH), 6:2 fluorotelomer sulfonic acid (6:2FTSA), perfluorohexanoic acid (PFHxA), perfluorohexanesulfonic acid (PFHxS), perfluorotridecanoate acid (PFTriDA), perfluorotetradecanoic acid (PFTeDA), aqueous film-forming foams (AFFFs), ammonium perfluorooctanoic acid ammonium salt (APFO), 6:2 chlorinated polyfluorinated ether sulfonate acid (F-53B), perfluoro acetic acid (cC6O4). In addition, keywords of some terms used to describe toxicity include oxidative stress, reproductive toxicity, genotoxicity, developmental toxicity, immunotoxicity, lifespan effects, behavioral toxicity, neurotoxicity, and mixed toxicity were searched for. A review of the screened references resulted in the selection of 137 articles, of which 28 were published before 2014 and the rest were published from 2014 to present.

## 3. Factors Contributing to the Accumulation of PFASs in Invertebrates

Researchers have demonstrated that PFASs are bioaccumulative [22] and that they can bioaccumulate and biomagnify at higher nutrient levels [23,24]. The bioaccumulation of PFASs is directly related to the carbon chain length, and long-chain PFASs are more easily accumulated than short-chain [25,26]. The long-chain PFASs have longer carbon chains and functional group differences to increase their hydrophobicity, which results in higher bioaccumulation. Therefore, PFASs with sulfonate groups may be more toxic than PFASs with carboxyl groups. Interestingly, it was found that long-chain PFASs inhibit the bioaccumulation of short-chain PFASs in tissues, probably because long-chain PFASs compete for transporter proteins and protein binding sites [27]. On the other hand, carbon chain length is also an important factor affecting the toxicity of PFASs. Long-chain PFASs are generally more toxic than short-chain PFASs, and they have stronger bioaccumulation and greater potential for biomagnification [28]. For example, *Eisenia fetida* was exposed to PFBS, PFHxS, 6:2 fluorotelomer sulfonic acid (6:2FTSA), and PFOS for 56 days, and the results showed the difference in toxicity between them to be 6:2FTSA < PFBS < PFOS < PFHxS. Molecular experiments and transcriptomic analyses revealed that 6:2FTSA, unlike PFOS, PFBS, and PFHxS, does not cause significant changes in antioxidant enzyme activities at the molecular level; additionally, *Eisenia fetida* exposed to 6:2FTSA did not produce adverse transcriptomic effects, which suggests that 6:2FTSA is the least toxic [29]. Interestingly, the exposure of *Brachionus calyciflorus* to short-chain PFASs resulted in a decrease in body size compared to the long-chain, which increased body size [30]. Bioaccumulation is also related to the type of contaminant, salt concentration, biological species differences, and the presence of metal ions [31,32].

Researchers studied PFASs in fish, crabs, gastropods, and bivalves collected from Korea, and they found that PFOA and PFOS had the largest bioconcentration factor (BCF) values in crabs, in fish it was PFOS and perfluorodecanoic acid (PFDA), and in gastropods and bivalves it was PFHxS. This confirms a species-dependent bioaccumulation of PFASs, which may be caused by differences in food sources, feeding objects, uptake and excretion rates, and metabolism between species [31,33]. Taylor examined PFOS, PFOA, and PFHxS in Dusky Flathead (*Platycephalus fuscus*), Mulloway (*Argyrosomus japonicus*), and Giant Mud Crab *(Scylla serrata*), and found the highest mean concentration of PFOS accumulation; this demonstrated that PFAS concentrations were negatively correlated with animal size [34].

Salt concentration largely affects the accumulation of PFASs. On the one hand, high salt concentration affects the physicochemical properties of PFOS and increases the partitioning ratio of PFOS between sediment–water, which leads to an increase in the adsorption of PFOS in the sediment [35], while benthic macroinvertebrates live in and around the sediment, which can interact with the sediment and related pollutants [36]. Therefore, high salt concentrations increase the uptake of contaminants by aquatic organisms and indirectly increase the accumulation of PFOS in aquatic organisms. On the other hand, the bioavailability of pollutants to aquatic organisms varies with changes in salt concentration. Under 30 salinity unit incubation conditions, sea urchins exhibited the shedding of spines, oxidative stress, and DNA methylation [32]. It has also been shown that changes in salinity levels after PFOS exposure largely affect the hemolymph hemocyanin content and the activity of the gill respiratory metabolic enzyme cytochrome C oxidase (CCO) in *Eriocheir sinensis* [37]. Exposure of oysters living at different salt concentrations to PFOS, PFOA, PFDA, and perfluoroundecanoic acid (PFUnDA) revealed that PFUnDA had the highest bioaccumulation in oysters and PFOA had the lowest. The accumulation of PFASs increased with increasing salt concentration, suggesting that the carbon chain length of PFASs as well as salinity affect the bioaccumulation of PFASs in the Pacific oyster [38].

Additionally, the level of contaminants in the soil, soil characteristics, and the duration of exposure can affect the bioaccumulation of some terrestrial invertebrates. Earthworms (*Eisenia fetida*) were tested using five different soils that differed in pH, organic carbon content, soil texture, and contaminant content. Bioaccumulation factors (BAFs) were found to be significantly higher in industrially impacted soils, urban soils, and AFFF soils exposed for 28 days than in control soils. In addition, they found that all BAFs of earthworms from PFAA-affected soils were greater than 1, except for PFDS in industrial-affected soils. This implies that contamination of soil with PFASs not only enters the terrestrial food chain but also bioaccumulates to higher concentrations in earthworms [39].

It was also found that cations can affect the partition ratio of PFASs in the environment through cation bridging effects, electrostatic interactions, and metal–ligand bonding interactions, which in turn affect the extent of bioaccumulation of PFASs in the environment [40,41,42]. The BAFs of PFASs in *Daphnia magna* showed a linear decreasing trend with increasing Ca^2+^ and Na^+^ concentrations. This may be due to the fact that the concentrations of Ca^2+^ and Na+ affect the partition coefficient (Kp) of PFASs between proteins and water; the higher the Kp value, the lower the concentration of freely soluble PFASs, and the lower the bioaccumulation coefficient of PFASs in *Daphnia magna* [43]. It has also been shown that the partitioning of PFASs to sediments increased when Mg^2+^ and Ca^2+^ concentrations were elevated, which increased the bioaccumulation of *Lumbriculus variegatus*, *Elliptio complanata*, and *Physella acuta*. This may be due to the fact that cations in water neutralize the negative charges associated with the sediment surface, reducing the electrostatic repulsion between the anionic PFASs and the negatively charged sediment and promoting the adsorption of PFASs through electrostatic interactions [44].

## 4. Toxicity Studies of PFASs in Invertebrates

**Figure 2:**
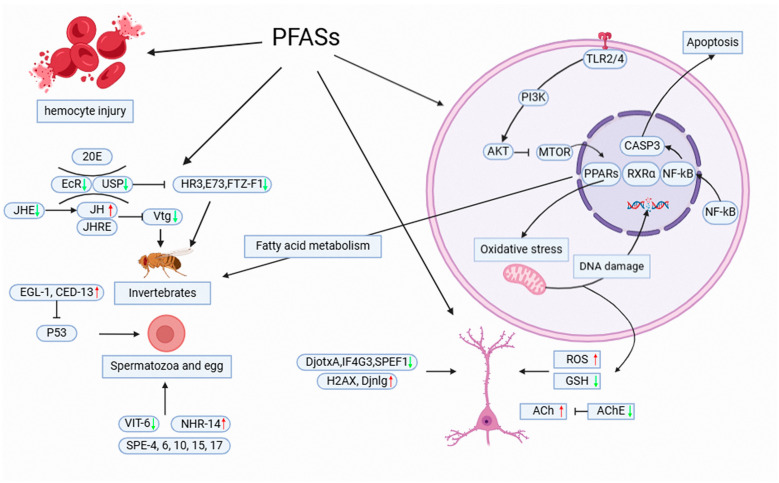
Schematic of the mechanism of toxicity of PFASs to organisms. PFASs cause oxidative stress, resulting in DNA breaks and reduced GSH activity, causing damage to nerve cells. PFAS exposure also affects genes related to nerve cell development and embryonic development. Reproductive organs and the generation and apoptosis of germ cells were also affected by the toxicity of PFASs. In addition, PFASs disrupt the function of blood cells and downregulate immune-related genes. The red up arrow indicates the gene is up-regulated, and the green down arrow indicates the gene is down-regulated.

Currently, toxicity studies in invertebrates have been partially conducted, not only limited to epigenetic characterization but also delving into their genetic changes in an attempt to explore the mechanisms of toxicity of PFASs. To this end, we have summarized them through toxicity classifications (Figure 2).

### 4.1. Invertebrate Lethal Concentration 50/Effect Concentration (LC50/EC50) for PFASs and Their Substitutes

The LC50/EC50 is used as an important parameter to measure the magnitude of toxicity of a toxicant to mammals and even humans. For this reason, this paper compiles the LC50/EC50 of PFASs on invertebrate studies in recent years (Table 1). From the results of this study, the LC50/EC50 values of different invertebrates exposed to the same pollutants for the same period of time varied greatly, which may be due to their different living environments and feeding patterns, etc. The LC50/EC50 values of the long-chained PFOA and PFOS were generally smaller than those of the short-chained PFBS, PFBA, etc., which indicated that the toxicity of long-chained PFAS was stronger, with PFOS being the most toxic. The LC50/EC50 values of long chains such as PFOS were generally smaller than those of short chains such as PFBS and PFBA, indicating that the toxicity of long-chain PFAS was stronger, with PFOS being the most toxic. This is worthy of attention because although the concentrations of pollutants in the experiment were much higher than those detected in the natural environment, the results caused by PFASs will be unpredictable with the prolongation of exposure time and the accumulation of food chain transmission. Both aquatic and terrestrial invertebrates have been poisoned by PFASs, not only limited to oxidative stress, reproductive toxicity, etc., but even death, which is a devastating blow to invertebrates and poses a great danger to the ecosystem.

### 4.2. Oxidative Stress

Oxidative stress, caused by an imbalance between oxidation and antioxidants in the body, can cause a range of diseases. PFASs produce a range of adverse effects by inducing oxidative stress, which may be caused by PFASs inducing ROS, which attack the DNA, resulting in breakage and nucleotide removal. This leads to a decrease in GSH activity. As an antioxidant that protects nerve cells, its reduced activity increases oxidative stress damage to nerve cells. Among terrestrial invertebrates, *Caenorhabditis elegans* is a good model organism, and it has been shown that PFOS exposure increases ROS levels in the body [71], inducing oxidative stress. Rijnders et al. found that a general inhibition of earthworm growth and an initial activation and then inhibition of the antioxidant stress in snails was related to PFAS concentrations and inferred from the results that snails may be more sensitive to PFOS and PFBA than other PFASs [72]. A general inhibition of earthworm growth and an initial activation and then inhibition of the antioxidant activities of superoxide dismutase (SOD), peroxidase (POD), catalase (CAT), and glutathione peroxidase (GSH-Px) were observed in PFOS-containing soils with controlled final concentrations of PFOS of 0, 10, 20, 40, 80, and 120 mg/kg. In addition, it was observed that glutathione (GSH-Px) content decreased and malondialdehyde (MDA) content increased during the exposure period [64,73]. A significant enhancement of SOD, POD, CAT, and malondialdehyde (MDA) activities was also found in earthworms living in soils containing chlorinated polyfluoroalkyl ether sulfonic acid (F-53B) [65]. In the study of *Nereidae*, which is also a member of the annelid phylum, it was also found that PFOS induced ROS production, which caused oxidative stress, resulting in changes in various oxidative stress indices and, finally, oxidative damage [37]. Exposure of another annelid, Lumbriculus variegatus, to the acids PFOA, PFOS, and PFDA resulted in a significant increase in MDA and CAT [74].

In addition, PFOA is equally widely used. One study found that earthworms exposed to PFOA also had increased MDA levels and activities of SOD, CAT, POD, and glutathione S-transferase (GST). A similar decrease in the activities of these antioxidant enzymes occurred with increasing exposure time [75]. The ability of PFOA to induce oxidative stress in earthworms is also illustrated by the fluctuations in SOD and CAT in the study of Wang et al. [76]. These are similar to the toxicity produced by PFOS exposure, and their mechanisms of action may be similar. In a recent study in the spotted cockroach, PFOA exposure increased head and midgut SOD, GSH-Px, and CAT activities but decreased fat body activities. PFOA also significantly increased GST activity and decreased GSH levels in the head, midgut, and fat body [77]. Culture of *Drosophila* with PFOA reveals that PFOA exposure produces oxidative stress, including changes in GSH and CAT activity [78]. In a short-chain study, PFBS, PFHxS, and 6:2FTSA were applied to earthworms exposed to PFOA, which produced excessive ROS. PFBS and PFHxS promoted SOD and CAT activities as well as increased MDA levels, whereas none of the 6:2FTSA had significant effects [29].

Among aquatic invertebrates, *Daphnia magna* is a classical model animal often used to assess the toxicity of various pollutants. High PFOS exposure significantly inhibits their GST activity, and their antioxidant system is significantly inhibited by short-term exposure to PFOS, which was experimentally demonstrated to be disrupted in *Daphnia magna* by long-term exposure to PFOS [79]. In a study on the offspring of *Daphnia magna*, it was found that exposure to high concentrations of PFOS resulted in a significant increase in GST levels in the offspring from the second generation to the fourth generation, which also indicated that PFOS causes an increase in oxidative stress in *Daphnia magna* and its offspring, suggesting that PFOS adversely affects the offspring of *Daphnia magna* [80]. *Dugesia japonica* also induced oxidative stress by PFOA after 10 days of exposure to PFOA, which was manifested by lipid peroxidation, significant elevation of glutathione S-transferase (GST), and a significant decrease in glutathione peroxidase (GSH-Px) activity [81]. It has been shown that exposure of *Mytilus edulis* to PFOA induces oxidative stress, with a decrease in CAT and SOD activities and a significant increase in GPx activity [82]. PFHxA, 6:2 FTA, PFUnDA, PFDoA, PFTriDA, and PFTeDA were evaluated using *Mytilus galloprovincialis* and found that in digestive glands, catalase activity increased and total antioxidant capacity decreased; additionally, the gill antioxidant enzyme activity was inhibited and oxidative stress increased [83]. Meanwhile, enzyme activation of CAT and SOD was observed in green mussels at lower exposure concentrations of PFOA and PFOS (0–100 μg/L), along with a decrease in GSH content. ROS production under PFOA and PFOS exposure was demonstrated to be dose-dependent, increasing with exposure concentration. At higher exposure concentrations (100–10,000 μg/L), both SOD and GSH-Px activities decreased [37,84,85]. In contrast, the activities of SOD and CAT fluctuated after PFOS exposure in sea urchins, with SOD activity increasing and then decreasing over time, and CAT following the opposite trend; this enhanced SOD activity may oxidize CAT and lead to a decrease in activity [32].

### 4.3. Neurobehavioral Toxicity

Acetylcholinesterase (AChE) is a key enzyme in biological nerve conduction between cholinergic synapses. This enzyme can degrade acetylcholine, terminate the excitatory effect of neurotransmitters on the postsynaptic membrane, and ensure the normal transmission of nerve signals in living organisms. The use of AChE activation is used as an indicator of neurological dysfunction for evaluation. Exposure to PFASs causes a reduction in the activity of AChE, resulting in an increase in the content of ACh and overexcitation of nerve cells, and also affects the expression of genes related to the development of nerve cells, resulting in the under-development of nerve cells and a reduction in branching, which in turn leads to inhibiting the normal activities of life. It has been shown that *Daphnia magna* significantly inhibits cholinesterase (ChE) in response to prolonged exposure to PFOS [79]. Jeong et al. found a significant increase in AChE activity in a study of the offspring of *Daphnia magna* exposed to PFOS. In a multigenerational response to discontinuous exposure, *Daphnia magna* was found to upregulate AChE during exposure and downregulate it during non-exposure [80]. Using green fluorescent protein (GFP)-tagged transgenic *Caenorhabditis elegans* nematodes, researchers found that PFOS exposure resulted in downregulation of the expression of the chemoreceptor gene gcy-5 in ASE chemosensory neurons [86]. By exploring the of PFOS on different neuron types, it was found that dopaminergic (DA) neurons were the most sensitive to PFOS, followed by gamma-aminobutyric acid (GABA)-ergic, serotonergic, and cholinergic neurons, and that the damage to DA was concentration- and time-dependent. It was also demonstrated that GSH alleviated the damage caused by PFOS [87]. It has been shown that chronic exposure of earthworms to PFOS and PFOA altered calcium homeostasis-related genes, with upregulation of NCS-2 and SSPO and downregulation of CANB2 and DUOX2, as well as altering the expression of genes related to neuronal development, such as the upregulation of H2AX, ANK1, and AT1A2, and downregulation of IF4G3, BTG-1, TSSK1, and DYH3 [88,89]. Several other studies have also shown that PFOA exposure reduces head acetylcholinesterase activity in *Nauphoeta cinerea* [77], and PFOA-induced behavioral deficits persist after cessation of exposure [90]. In a study of the triploid whirligig *Dugesia japonica*, PFOA produced damage to the ventral nerve cord, while PFOS mainly affected the cranial ganglia and caudal nerves [37]. Detection of neurodevelopment-related genes in *Dugesia japonica* exposed to PFOS revealed that DjotxA, DjotxB, DjFoxD, and DjFoxG were downregulated, while Djnlg was upregulated. The brain shape of the experimental group was shown to be abnormal by immunofluorescence, with smaller head ganglia and reduced nerve fiber density and brain branching, and the experimental results showed that AChE first increased and then decreased with exposure time [91].

The toxicity of PFASs to invertebrates is often accompanied by changes in their behavior that are inextricably linked to their neurotoxic effects. In existing studies, PFOA exposure was found to cause abnormal foraging behavior in *Drosophila* larvae, which is manifested by the larvae moving slowly and away from food [92]. Under PFOA exposure, spotted *Nauphoeta cinerea* showed a significant decrease in locomotor activity and a significant increase in the time of cessation of immobility [77]. At PFOS concentrations higher than 10 mg/L, damselfly larvae activity was reduced, the ability to escape from simulated predators was reduced, and foraging efficiency was reduced [93]. It has been shown that *Caenorhabditis elegans* exposed to PFOS at a concentration of 20 μmol results in reduced locomotor behavior, including forward movement, body-bending, and head-bobbing [86]. It has also been shown that PFOS exposure also causes offspring to exhibit the same locomotor deficits as their parents, such as a reduction in the frequency of body-bending and head-bobbing [63]. Exposure of Lumbriculus variegatus to PFOA, PFOS, and PFDA for 12 days results in escape behaviors that indicate retardation [74]. In contrast, a significant avoidance behavior was also found in earthworms affected by PFOS and F-53B, as evidenced by the continuous escape of earthworms from soil to control soil [65]. In another test, no significant adverse reaction of *Daphnia magna* was detected at PFOS concentrations below 30 mg/L. Almost all *Daphnia magna* remained immobile when the concentration was higher than 150 mg/L. Increasing PFOS concentrations stimulated the heartbeat but suppressed heart rate in high concentrations [79]. Gammarus feeding was affected and food consumption was reduced after exposure to 100 ng/L PFBA [94]. *Faxonius immunis* were similarly exposed to PFAS and resulted in reduced foraging behavior [95].

### 4.4. Developmental and Reproductive Toxicity

In several recent studies, PFASs were found to have severe toxicity on developmental reproduction. Adult *Drosophila* flies exposed to 500 nM PFOA lost significant weight compared to controls, while PFOA had a strong effect on *Drosophila* larval development, as evidenced by impaired plumage change, inability of most larvae to transition from the first to the second instar, and polyphasic lethality [92]. PFBA was also found to affect normal development in the beet armyworm moth. The weight of 2nd~5th instar larvae increased after exposure, and the time to pupation and time to plumage were shortened [96]. European honey bee (*Apis mellifera*) larvae also completely stopped developing at the lowest exposure concentration of PFOS, 0.02 mg/L, and the other exposure concentrations also reduced the body weights of the adult moths [97]. In crustaceans, the presence of ecdysteroids (20-hydroxyecdysone, 20E) and juvenile-preserving hormone (JH) allows for EcR receptors and USP to bind and upregulate the induction of developmental and molting-related genes [98]. Exposure to PFASs downregulates the gene expression of EcRA and EcRB, as well as USP, and JHE—an enzyme that hydrolyzes JH—is also downregulated. The upregulation of JH leads to the downregulation of Vtg genes, which are regulated by the binding of JH and JHRE [99] and affect yolkgenesis and embryonic development. In contrast, body weight differences during development are PPAR-mediated lipid-metabolism-related. In *Daphnia magna*, downregulation of genes involved in developmental and reproductive processes was observed after exposure to 10 μm (5 mg/L) and 25 μm (12.5 mg/L) PFOA and 25 μm (10.35 mg/L) PFOS, including Vtg2, VASA, EcRA, EcRB, USP, JHE, HR3, FTZ-F1, E74, and E75 [100]. PFOS in *Tigriopus japonicus* also caused a delay in the growth time of the unsegmented larvae to the adult stage [101]. PFOA also resulted in slowed growth and developmental delay in *Hyalella azteca*, with a proportion unable to reach sexual maturity [53]. *Caenorhabditis elegans* were exposed to PFBA, PFHxA, PFOA, PFBS, PFHxS, PFOS, 1H,1H, 2H, and 2H-perfluorooctanesulfonamidoacetic acid (NEtFOSAA), 6:2 FTSA, perfluorooctanesulfonamide (PFOSA), and hexafluoropropylene oxide dimeric acid (HFPO-DA); it was found that all of these PFASs exhibited a significant inhibition of their growth in the adult stage [102]. Treatment of *Daphnia* at all dilute concentrations of aqueous film-forming foams (AFFFs) revealed developmental disorders manifested by a significant reduction in body length [103]. Interestingly, compared to exposure to long-chain PFOA, the rotifer *Brachionus calyciflorus* exposed to short-chain PFBA, PFPeA, and PFHxA showed a significant increase in body size, suggesting that the ecological effects of the short-chain PFCAs are different from those of long-chain [30].

PFASs have been found to have toxic effects on reproductive organs as well as on the conception of offspring in both reproductive aspects. In terms of reproductive toxicity, PFASs first accumulate in organs and cause toxicity in them, and have secondary effects on germ cell (sperm and egg) production and apoptosis. This is exacerbated by the upregulation of EGL-1 and CED-13, which increase the activity of apoptosis-initiating protein p53 [104], exacerbating apoptosis. By drinking PFOS-containing sugar water, it was found that the onset of ovary size in bumblebee *Bombus terrestris* was drastically reduced, with a reduction in ovary length of more than 50% [59]. Preconceptional exposure of *Drosophila melanogaster* to 2 ng of PFOS resulted in a significant reduction in megalin transcript levels, a decrease in egg production, the retardation of offspring development, as well as altered expression of the *Drosophila melanogaster* insulin-like peptide (DILP) [105]. *Enallagma cyathigerum*, also an insect, showed decreased egg-hatching success, delayed larval development, and increased mortality after exposure to PFOS [106]. A study evaluating sperm changes in *Caenorhabditis elegans* nematodes exposed to 0.001, 0.01, and 0.1 mmol/L PFOS or PFOA for 48 h found that exposure induced reproductive toxicity in male nematodes, resulting in reduced brood size, lower germ cell counts, reduced spermatocyte size and viability, and increased sperm malformations. Further studies found that this damage was caused by the disruption of spermatogenesis, including mitotic and meiotic progression, formation of membranous organelles (MO), fusion of MO and the plasma membrane (PM), and pseudopods, which were inseparably linked to changes in the SPE-4, SPE-6, SPE-10, SPE-15, SPE-17, and fer-1 genes [107]. Female nematodes exposed to PFOA and PFOS, on the other hand, were responsible for reduced egg production and progeny [108]. In addition, PFOS exposure resulted in significantly delayed gonadal development in *Caenorhabditis elegans* nematodes[71]. It was also found that exposure of *Caenorhabditis elegans* to HFPO-DA 1.5–4 g/L for 48 h resulted in developmental delay, and a statistically significant delay in progeny production was observed, the extent of which correlated with the concentration of HFPO-DA, with higher concentrations resulting in later egg laying [109]. It has also been shown that, in *Caenorhabditis elegans*, germ cell apoptosis after PFBS exposure occurs, with an upregulation of pro-apoptotic genes EGL-1 and CED-13, a significant reduction in egg laying and brood size, downregulation of reproduction-related genes VIT-6 (vitellogenin 6, encoding vitellogenin), and upregulation of NHR-14 (nuclear hormone receptor 14, encoding estrogen receptor) [61]. In addition, cC6O4 completely inhibited the reproduction of *Eisenia fetida* at a concentration of 1390 mg/kg d. w. At a concentration of 2.79 mg/kg d. w., the yield reduction in offspring was 27% [66].

In aquatic environments, PFOS at 8 mg/L significantly inhibited most of the reproductive behaviors of *Daphnia magna*, higher PFOS concentrations delayed the production of offspring, and the prolongation of *Daphnia* reproduction was more or less correlated with toxicity [49]. Jeong et al. investigated the multigenerational toxicity of PFOS on the number of offspring and the time to first brood. The reproductive ability of *Daphnia magna* was inhibited during the first generation after continuous exposure to PFOS [80]. In another study on *Daphnia magna*, it was observed that PFOS at concentrations higher than 1 μM and PFOA at concentrations higher than 24 μM resulted in inhibition of reproduction [110]. It was also found that the number of *Daphnia magna* first broods first decreased, and then rose and fell with increasing concentrations. At a concentration of 2 mg/L, *Daphnia magna* continuously exposed to PFOS may have adapted to the environment, resulting in a partial rise in population [79]. *Daphnia magna* exposed to AFFFs, which are also PFASs, have been found to have a decrease in reproduction capacity with increasing exposure concentrations until complete loss occurs [103]. Perfluoroethylcyclohexane sulfonate (PFECHS), a member of the perfluoroalkyl sulfonate family, also causes significant downregulation of the vitellogenin-related gene (Vtg1) in *Daphnia magna* [111]. Unlike *Daphnia magna*, which inhabits the pelagic layer, exposure of the Daphnia carinata to high concentrations of PFOA and PFOS was also found to significantly affect the time to first brood and reduce the number of offspring surviving. At PFOS concentrations greater than 1 mg/L, reproduction was even completely inhibited and an absence of offspring occurred [51]. The rotifer *Brachionus calyciflorus* resulted in a reduction in egg size after exposure to both PFOS and PFOA, with PFOS significantly reducing the reproductive rate of the rotifer [30]. Calculating the hatchability of resting eggs exposed to PFOA/PFOS during egg incubation, a significant reduction in hatchability occurred when the PFOA concentration was higher than 0.25 mg/L, whereas a significant reduction in hatchability occurred when the PFOS concentration was higher than 2 mg/L [112]. Paracentrotus lividus induces malformations in its embryos by accumulating in large quantities in the gonads after exposure to PFOA [113].

### 4.5. Immunogenetic Toxicity

Invertebrates do not have an adaptive immune system and rely on the innate immune response to defend against pathogens, and blood cells are an important component of this innate immunity. The immunotoxicity of PFASs is achieved by disrupting the function of blood cells as well as the regulation of immune factors due to the downregulation of immune-related genes. *Ruditapes philippinarum* showed significant disruption of the subcellular structure of blood cells under PFOA exposure, and the phagocytosis of blood cells increased and then decreased with the duration of exposure [114]. Researchers also found that immune-related genes and pathways were altered, such as the PI3K-Akt-mTOR, NF-KB, and IL-17 signaling pathways and genes encoding TLR2/4/6, MyD88, and NFkB1 being significantly upregulated [115]. Using transcriptome analysis, a large number of immune molecules were found to be significantly downregulated in *Ruditapes philippinarum* exposed to 20 mg/L PFOS. This includes C1q—involved in innate immunity—as well as the glycoprotein-binding family, which plays an important role in immune regulation, among others [116]. Exposure experiments using PFOS, PFOA, PFNA, and PFDA on the Perna viridis also revealed a decrease in the number of hemocyte activities and a similar decrease in phagocytosis, and also found that these immunotoxicities were reversible [117]. *Eriocheir sinensis* was exposed to 0, 0.01, 0.1, 1.0, and 10 mg/L PFOS on days 1, 4, 7, 14, and 21, and significant reductions in the number of hemocytes were observed, as well as significant reductions in the activity of the phenoloxidase enzyme, which is important for natural immunity [118]. Exposure of *Ruditapes philippinarum* to short-chain C6O4 at concentrations of 0.1 μg/L and 1 μg/L similarly revealed upregulation of genes encoding C-type lectins, complement C1q-like proteins, and interferon-inducible GTPase 1-related genes [119].

### 4.6. Genotoxicity

Genotoxicity refers to damage to genetic material (e.g., chromosomes or DNA) by physical or chemical agents. It has been shown that both PFOA and PFOS cause DNA damage in *Dugesia japonica*, which is manifested by an increase in tail DNA and tail moment content [120,121]. Exposure of green mussels (Perna viridis) to concentrations of 0.1, 1, 10, 100, and 1000 μg/L PFOS, PFOA, PFNA, and PFDA revealed DNA fragmentation and chromosome breakage by comet assay, MN assay, and DNA diffusion assay [58]. For Daphnia carinata exposed to PFOA and PFOS, significant DNA fragmentation was similarly found to occur in PFOA and PFOS at concentrations of 1 mg/L and 10.0 mg/L [51]. The treatment of the *Aporrectodea caliginosa* with different concentrations of PFOA and PFOS and the extraction of earthworm luminal cells for comet assay revealed that PFOS and the mixture of PFOS + PFOA induced DNA damage, which was absent in PFOA, and genotoxicity increased with increasing concentrations [122]. In contrast, PFOS did not cause DNA damage in the *Eisenia fetida* exposure experiment, whereas PFOA increased DNA migration [123]. A study found that all concentrations of PFOS caused DNA damage in *Eisenia fetida*. DNA damage was found to be caused by oxidative stress, and ROS caused DNA damage by causing DNA strand breaks, removing nucleotides, and modifying nucleotide bases [73]. PFOS and PFOA have also been shown to cause DNA damage, with PFOS being more toxic than PFOA [69].

### 4.7. Lifetime Impact

PFASs reduce invertebrates’ lifespan ordinarily. It has been shown that PFASs activate DAF-16 by inhibiting DAF-2 and promote the movement of DAF-16 from the cytoplasm into the nucleus, thereby extensively regulating the expression levels of downstream genes related to stress resistance, detoxification, and metabolism [124]. Researchers have found a shortening effect of PFOA on the lifespan of male *Drosophila* but no significant effect on the lifespan of adult females [92]. It has been found that exposure to 0.2–200 μM PFOS for 50 h shortens lifespan in a concentration-dependent manner in *Caenorhabditis elegans* nematodes. Lifespan has been reported to be affected by mutations in the daf-16, daf-2, or age-1 genes, which are associated with the insulin/IGF-1 signaling pathway (IIS) in transgenic *Caenorhabditis elegans* nematodes [125]. It has also been shown that the lifespan of *Caenorhabditis elegans* nematodes is significantly reduced in the presence of high concentrations of PFOS ≥ 1.0 μM [63], which demonstrates that PFOS exposure accelerates senescence and shortens the lifespan of the animals. Seyoum et al. exposed the entire lifespan of *Daphnia magna* to PFOS, and found that the control daphnia’s lifespan was 45 days; *Daphnia magna* exposed to 1 μM and 10 μM PFOS and all concentrations of PFOA had a lifespan of 39 to 41 days; and *Daphnia magna* exposed to 25 μM PFOS had a severely reduced lifespan of 23 days [100]. Exposure of the rotifer *Brachionus calyciflorus* to both 2.0 mg PFOA/L and PFOS revealed reductions in their survival times [30].

## 5. Mixed Toxicity

It is well-known that water bodies as well as soils contain numerous chemicals that can act as initiators or as antagonists interacting with PFASs to produce unknown toxicity. In the example of metal ions, it has been shown that the presence of PFOS significantly affects the toxicity of Cu and Cd in earthworms, exacerbating oxidative stress [126,127]. The presence of PFASs was also found to increase the uptake of Cd, Zn, Ni, Pb, and Cu from soil by earthworms in a study by Zhao et al., where PFASs and metals interacted with each other to affect their bioaccumulation and subcellular distribution in earthworms [128]. Another study also illustrated that exposing earthworms to soil containing PFOA, arsenite (As(III)) caused higher oxidative stress and increased arsenic bioaccumulation in earthworms, which in turn reduced PFOA bioaccumulation [68]. A similar situation was found in artificial soils exposed to PFOA and four arsenic species—arsenite (As(III)), arsenate (As(V)), monomethylarsenate (MMA), and dimethylarsenate (DMA)—causing greater toxicity to earthworms that was not limited to growth inhibition and oxidative stress, and demonstrating that the type of As present in the artificial soils has a greater effect on co-toxicity [129]. Co-exposure of PFOA with organic As resulted in antagonistic responses, while co-exposure with inorganic As resulted in synergistic responses [129]. Along with the metal ion interactions, studies have also found the effects of pH and the co-treatment of Zn and PFOS on *Caenorhabditis elegans* caused the 24 h-EC50 value of Zn metal to decrease with a decreasing pH or an increasing exposure concentration of PFOS [62]. In a study on the toxicity of co-exposure of PFOS and suspended sediment (SPS) on *Corbicula fluminea*, it was found that there was a significant increase in bioaccumulation and oxidative stress, where indicators such as SOD and CAT activities were significantly increased. Co-exposure also causes histopathological alterations in the gonads and digestive glands, such as shrinkage of oocytes, loss of epithelial cells, and even inhibition of siphoning behavior in clams [130]. Combined exposure to polystyrene microplastics (MPs) and ammonium perfluorooctanoic acid ammonium salt (APFO) is found to have synergistic and antagonistic effects on their mixed toxicity, with APFO inducing an alteration in MP intestinal fullness in *Daphnia magna*. At high concentrations, the mixed toxicity of MPs and APFO elicits intestinal obstruction-mediated physiological and biochemical responses in *Daphnia magna* [131]. It was also found that lower concentrations of MPs adsorbed more PFOS, and that combined exposure to MPs and PFOS impeded the regeneration of the eyepatches of decapitated *Dugesia japonica*, resulting in severe DNA damage. Treatment with MPs and PFOS not only impaired the proliferation of neoblasts in *Dugesia japonica* but also impeded the regeneration and development of the nervous system [132]. Co-exposure of PFOA and nano-TiO_2_ caused a worse case of oxidative stress [133]. It also had a toxic effect on immune function and significantly reduced the total blood count, mitochondrial count, and lysosomal content [134]. It also interfered with the normal byssal line secretion process of *Mytilus coruscus*, resulting in damage to its foot structure, weakening byssal performance, and affecting its defense capability [135]. Co-exposure to 2,2′,4,4′-Tetrabrmodiphenyl ether (BDE-47) and PFOA exacerbates oxidative stress in *Mytilus galloprovincialis* [136]. Thus, it is expected that the toxicity of PFASs in the environment may be far greater, and it is urgent to improve the laws and regulations.

## 6. Conclusions

PFASs have become global pollutants and have been found in many environments and organisms. Many long-chain PFASs have been banned but their use has resulted in their accumulation in the environment where they are not readily degradable, and where they will pose a serious threat to organisms in the environment for a long time to come. According to the existing literature, PFASs are already known to be reproductively toxic, neurotoxic, developmentally toxic, immunotoxic, and metabolically toxic. They enter the environment through daily production activities, first accumulating in plants and invertebrates, then bioaccumulating through the food chain, and ultimately completing their accumulation in organisms, where they cause a range of adverse effects, including reproductive degradation, developmental delay, and neurological damage in invertebrates. PFASs can also be combined with other pollutants in the environment to form a combination of toxicities, deepening the toxicity of PFASs. The bioconcentration capacity and toxicity of PFASs are strongly related to their chain length, which is generally positively correlated. Of the invertebrates tested, aquatic invertebrates appeared to be more sensitive than terrestrial invertebrates, possibly due to differences in living environments and the fact that contaminants are more likely to be deposited in the aquatic environment. From the data, *Chironomus dilutus* was the most vulnerable to PFASs, which harmed it at very low concentrations.

PFASs have now spread all over the world, with traditional long-chain PFASs being replaced by emerging short-chain PFASs. The ecological impact of short-chain PFASs needs to be further investigated, especially in terrestrial invertebrates where gaps still exist. Study of the combined exposure toxicity of multiple PFASs at environmentally relevant concentrations is also critical. In the future, it is necessary to strictly control the production, use, transportation, and disposal of PFASs, reduce the direct or indirect entry of PFASs into the environment, emphasize the remediation of ecological pollution, and strengthen the research on the degradation of PFASs and their substitutes in order to reduce the environmental risks of PFASs.

## Figures and Tables

**Table 1:** EC50/LC50 values for target PFASs and some alternatives.

Species	PFAS Type	LC50/EC50 (95% Confidential Interval)	Test *Duration*	Reference
Aquatic invertebrate				
*Brachionus calyciflorus*	PFOS	61.8 mg/L	24 h–LC50	[30]
	PFOA	150 mg/L	24 h–LC50	
*Chironomus dilutus*	PFOS	0.0075 (0.0066–0.0085) mg/L	16 d–LC50	[45]
		20 (15–26) mg/L	6 d–LC50	
*Chydorus sphaericu*	PFBA	462.32 (443.06–481.37) mg/mL	48 h–EC50	[46]
	PFOA	116.77 (50.52–142.85) mg/mL	48 h–EC50	
	PFNA	27.84 (21.81–32.49) mg/mL	48 h–EC50	
	PFDA	45.24 (26.22–63.75) mg/mL	48 h–EC50	
	PFUnA	19.18 (12.41–23.69) mg/mL	48 h–EC50	
	PFDoA	28.25 (20.88–49.74) mg/mL	48 h–EC50	
*Daphnia magna*	PFBA	181.5 (180–183.21) mg/mL	48 h–EC50	[46]
	PFOA	211.59 (184.68–255.48) mg/mL	48 h–EC50	
	PFNA	151.29 130.46–181) mg/mL	48 h–EC50	
	PFDA	163.48 (143–177.36) mg/mL	48 h–EC50	
	PFUnA	133.13 (91.95–184.46) mg/mL	48 h–EC50	
	PFDoA	79.22 (60.18–98.26) mg/mL	48 h–EC50	
	PFBS	2183 (1707–3767) mg/L	48 h–LC50	[47]
	PFNA	43.42 mg/L	48 h–EC50	[48]
	PFOS	23.41 mg/L	48 h–EC50	
	PFOA	476.5 (375.72–577.72) mg/L	48 h–EC50	[49]
	PFBA	5251 (3889–6614) mg/L	48 h–EC50	[50]
	PFHxA	1048 (802–1294) mg/L	48 h–EC50	
	PFOA	239 (190–287) mg/L	48 h–EC50	
	PFOS	8.8 (6.4–11.6) mg/L	48 h–LC50	[51]
	PFOA	78.2 (54.9–105) mg/L	48 h–LC50	
*Dugesia japonica*	PFOS	34 (30–38) mg/L	24 h–LC50	[52]
		27 (24–31) mg/L	48 h–LC50	
		26 (23–29) mg/L	72 h–LC50	
		23 (20–25) mg/L	96 h–LC50	
	PFOA	352 (331–374) mg/L	24 h–LC50	
		345 (325–366) mg/L	48 h–LC50	
		343 (324–364) mg/L	72 h–LC50	
		337 (318–357) mg/L	96 h–LC50	
*Hyalella azteca*	PFOA	113 (103–124) mg/L	7 d–LC50	[53]
		87.8 (79.8–96.5) mg/L	14 d–LC50	
		70.2 (63.6–77.5) mg/L	21 d–LC50	
		57.5 (51.3–64.5) mg/L	28 d–LC50	
		55.1 (49.0–62.0) mg/L	35 d–LC50	
		51.5 (45.6–58.1) mg/L	42 d–LC50	
	PFOS	15 (13– 18) mg/L	42 d–LC50	[45]
*Lampsilis siliquoidea*	PFOS	16.5 (8.0–33.9) mg/L	24 h–EC50	[54]
		17.7 (7.2–43.5) mg/L	48 h–EC50	
	PFOA	164.4 (116.0–232.8) mg/L	24 h–EC50	
		162.6 (130.6–202.3) mg/L	48 h–EC50	
*Ligumia recta*	PFOS	13.5 (5.7–31.8) mg/L	24 h–EC50	
		17.1 (9.4–31.1) mg/L	48 h–EC50	
	PFOA	161.0 (135.8–191.0) mg/L	24 h–EC50	
		161.3 (135.0–192.7) mg/L	48 h–EC50	
*Moina macrocopa*	PFOA	199.51 (153.89–245.13) mg/L	48 h–EC50	[49]
*Moina micrura*	PFOS	549.6 (407.2–743.9) μg/L	48 h–LC50	[55]
	PFOA	474.77 (350.4–644.5) μg/L	48 h–LC50	
*Mytilus galloprovincialis*	PFOS	1.07 (1.06–1.08) mg/L	48 h–LC50	[56]
	PFOA	9.98 (9.6–10) mg/L	48 h–LC50	
*Neocaridina denticulate*	PFOS	57 (43–75) mg/L	48 h–LC50	[52]
		20 (17–24) mg/L	72 h–LC50	
		10 (9–12) mg/L	96 h–LC50	
	PFOA	712 (663–764) mg/L	48 h–LC50	
		546 (502–594) mg/L	72 h–LC50	
		454 (418–494) mg/L	96 h–LC50	
*Paracentrotus lividus*	PFOS	20 (15.8–25.3) mg/L	72 h–EC50	[57]
	PFOA	110 (99.2–121.9) mg/L	72 h–EC50	
*Perna viridis*	PFOS	33 (29–37) μg/L	7 d–EC50	[58]
	PFOA	594 (341–1036) μg/L	7 d–EC50	
	PFNA	195 (144–265) μg/L	7 d–EC50	
	PFDA	78 (73–84) μg/L	7 d–EC50	
*Siriella armata*	PFOS	6.9 (6.8–7.0) mg/L	72 h–EC50	
	PFOA	15.5 (13.0–18.6) mg/L	72 h–EC50	[57]
Terrestrial invertebrate				
*Bombus terrestris*	PFOS	1.01 (0.6–1.8) mg/L	11 weeks–LC50	[59]
*Caenorhabditis elegans*	PFOS	4.522 mg/L	24 h–LC50	[60]
	PFOA	22.655 mg/L	24 h–LC50	
	PFBS	238.28 (187.26–302.8) μg/mL	48 h–LC50	[61]
	PFOA	0.58 (0.45–0.66) μg/mL	48 h–LC50	
	PFBS	481.5 mg/L	48 h–LC50	[62]
	PFOS	1.57 (1.36–1.8) μg/mL	48 h–LC50	[63]
*Eisenia fetida*	PFOS	32.40 μg/cm^2^	24 h–LC50	[64]
		26.28 μg/cm^2^	48 h–LC50	
	PFOA	21.34 μg/cm^2^	24 h–LC50	
		14.95 μg/cm^2^	48 h–LC50	
	PFOS	1302.57 mg/kg	7 d–LC50	[65]
		913.3 mg/kg	14 d–LC50	
	F-53B	1118.52 mg/kg	7 d–LC50	
		816.06 mg/kg	14 d–LC50	
	cC6O4	10.4 mg/kg	56 d–EC50	[66]
	PFOS	405.3 (373.8–439.5) mg/kg	7 d–LC50	[67]
		365.4 (333.6–400.2) mg/kg	14 d–LC50	
	PFOA	1307 (1236.1–1394.5) mg/kg	7 d–LC50	
		1000.8 (926.2–1081.5) mg/kg	14 d–LC50	
	PFOA	812 mg/kg	14 d–LC50	[68]
	PFOS	478.0 mg/kg	14 d–LC50	[69]
	PFOA	759.6 mg/kg	14 d–LC50	
*Folsomia candida*	PFOS	130 (101–167) mg/kg	28 d–LC50	[70]
*Oppia nitens*	PFOS	65 (59–72) mg/kg	28 d–LC50	[70]
*Physa acuta*	PFOS	271 mg/L	24 h–LC50	[52]
		233 (226–241) mg/L	48 h–LC50	
		208 (197–219) mg/L	72 h–LC50	
		178 (167–189) mg/L	96 h–LC50	
	PFOA	856 (768–954) mg/L	24 h–LC50	
		732 (688–779) mg/L	48 h–LC50	
		697 (661–735) mg/L	72 h–LC50	
		672 (635–711) mg/L	96 h–LC50	

## Data Availability

Data are contained within the article.
